# Precise and selective sensing of DNA-DNA hybridization by graphene/Si-nanowires diode-type biosensors

**DOI:** 10.1038/srep31984

**Published:** 2016-08-18

**Authors:** Jungkil Kim, Shin-Young Park, Sung Kim, Dae Hun Lee, Ju Hwan Kim, Jong Min Kim, Hee Kang, Joong-Soo Han, Jun Woo Park, Hosun Lee, Suk-Ho Choi

**Affiliations:** 1 Department of Applied Physics and Institute of Natural Sciences, College of Applied Science, Kyung Hee University, Yongin 446-701, Korea; 2 Department of Biochemistry and Molecular Biology, College of Medicine, Hanyang University, Seoul 133-791, Korea; 3 Department of East-West Medical Science, Graduate School of East-West Medical Science, Kyung Hee University, Yongin 446-701, Korea

## Abstract

Single-Si-nanowire (NW)-based DNA sensors have been recently developed, but their sensitivity is very limited because of high noise signals, originating from small source-drain current of the single Si NW. Here, we demonstrate that chemical-vapor-deposition-grown large-scale graphene/surface-modified vertical-Si-NW-arrays junctions can be utilized as diode-type biosensors for highly-sensitive and -selective detection of specific oligonucleotides. For this, a twenty-seven-base-long synthetic oligonucleotide, which is a fragment of human DENND2D promoter sequence, is first decorated as a probe on the surface of vertical Si-NW arrays, and then the complementary oligonucleotide is hybridized to the probe. This hybridization gives rise to a doping effect on the surface of Si NWs, resulting in the increase of the current in the biosensor. The current of the biosensor increases from 19 to 120% as the concentration of the target DNA varies from 0.1 to 500 nM. In contrast, such biosensing does not come into play by the use of the oligonucleotide with incompatible or mismatched sequences. Similar results are observed from photoluminescence microscopic images and spectra. The biosensors show very-uniform current changes with standard deviations ranging ~1 to ~10% by ten-times endurance tests. These results are very promising for their applications in accurate, selective, and stable biosensing.

Silicon nanomaterials widely known as one of the most biocompatible materials have been successfully applied to various kinds of bionics fields such as imaging cells, biosensors, cancer theranostics, bio-integrated electronics, and drug deliveries^1^,^2^,^3^,^4^,^5^,^6^,^7^,^8^. Especially, the application of silicon nanowires (Si NWs) to biosensors have received much attention due to their possible detection of biological species such as DNAs, RNAs, proteins, virus, and others^9^,^10^,^11^,^12^,^13^,^14^,^15^. The unique structural properties of Si NWs enable them to serve as a building block for biological sensors by monitoring the change of electrical current. The binding of bio-species with the surface of Si NWs can bring about accumulation or depletion of charge carriers (negative electrons or positive holes), thereby affecting the variation of the electrical properties in Si NWs, which is more sensitive than in bulk Si because of high surface/volume ratio of Si NWs, resulting from their ultrathin diameter^11^,^12^,^14^.

Recently, there have been several reports on field-effect-transistor (FET)-based electrochemical DNA sensors employing conducting polymers and single planar Si NW^9^,^11^,^14^,^16^, that are label-free, and real-time-monitoring available. However, these sensors generally require large amount of DNA solution for the differential pulse voltammetry because they should be soaked in the solution for this technique. The planar-shape active region of the sensors limits their sensitivity. The single Si-NW FET sensors are fundamentally three-electrode structures, which requires complicated fabrication processes for the gating channels. Due to the single wire-structure, the responsivity and the absolute-current change stick around several tens of percent and order of nA, respectively. No such limitations exist in vertical Si-NW bundles because the sum of the current flowing through Si-NW arrays is much larger than the current in the single planar Si NW, and the noise current of Si NW arrays can be sufficiently ignored, resulting in great enhancement of the sensitivity and responsivity^17^. Graphene and carbon nanotubes have been also employed in FET-type biosensors, but the detection range of DNA is just down to 100 nM^18^, and the current response is in the orders of nA and μA^19^.

Here, we first report remarkable sensitivity and selectivity of the graphene/surface-modified-Si-NWs vertical-junction diodes in detecting the hybridization of oligonucleotides. Conventional nucleic-acid-based diagnostics by using real-time polymerise-chain-reaction facilities require large volume of space for set up, which makes it impossible for the technique to be realized into into portable devices and nano-scale information processing systems. In contrast, the approach for DNA diognostics by employing graphene/Si-NWs biosensors can extremely reduce them to be portable and integrable.

The principal processes and mechanism for the selective and precise DNA detection by graphene/Si-NWs vertical-junction diodes are described as follows. A probe sequence (p-ss oligonucleotide, tta gcg cgg agt tgg gag cgg gag tcg) and a target sequence, which is complementary to the probe (t-ss oligonucleotide, cga ctc ccg ctc cca act ccg cgc taa), and dummy single strand oligonucleotide (tct tgc aca agt tta aga ggg aaa gga) (d-ss oligonucleotide) are employed in this work^20^. The p-ss oligonucleotide is utilized as DNA acceptors for hybridization to t-ss oligonucleotide. The d-ss oligonucleotide having mismatched nucleotide sequences to those of p- and t-ss oligonucleotides is utilized for the negative control experiments. As shown in Fig. 1a, a solution containing p-ss oligonucleotide is dropped on the area without graphene in the devices, by which the surface of Si NWs is soaked, resulting in the decoration of Si NWs with p-ss oligonucleotide. The t- or d-ss oligonucleotide is then dropped on the devices. The t-ss oligonucleotide is well combined with the p-ss oligonucleotide on the surface of Si NWs because their nucleotide sequences are complementary with each other, but this cannot happen for d-ss oligonucleotide, as shown in Fig. 1b. Based on this mechanism, the graphene/Si NWs diodes are employed as biosensors for selectively and precisely detecting DNA-DNA hybridization. The electrical current of the biosensors is increased only when the t-ss oligonucleotide is dropped on the p-ss oligonucleotide-decorated devices. Photoluminescence (PL) microscopy and spectroscopy of the oligonucleotide-decorated Si NWs also proves the DNA-sensing mechanism.

## Results

### Fabrication of surface-modified Si NWs

**Figure 2:**
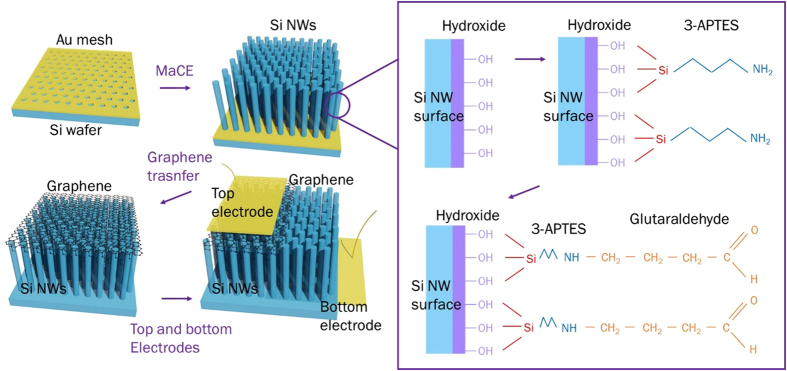
Schematic diagrams describing fabrication processes of graphene/Si-NWs biosensors. Schematic diagram of fabrication processes at each step: 1) preparing vertically-aligned Si-NW arrays by MaCE, 2) modifying the surface of Si NWs for sensing DNAs, 3) forming the vertical junction of graphene/surface-modified Si NWs, and 4) finally completing graphene/Si-NWs vertical-diode-type biosensors by depositing top/bottom metal contacts. These fabrication steps are detailed in the text.

**Figure 3:**
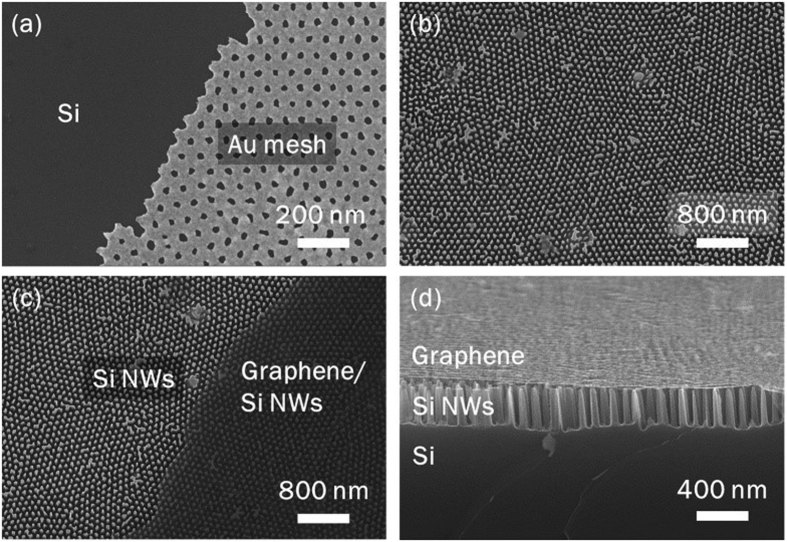
SEM images at each step for fabricating of graphene/Si-NWs biosensors. Planar SEM images of (**a**) Au mesh with hole arrays on the Si substrate, (**b**) Si-NW arrays prepared by MaCE, and (**c**) uniformly-transferred graphene on the top of Si NWs. (**d**) Tilted SEM image of graphene/Si-NWs biosensors, clearly showing the uniform contact between graphene and Si NWs.

The fabrication procedures of graphene/Si-NWs biosensors are schematically illustrated in Fig. 2. The Si NWs were fabricated by metal-assisted chemical etching (MaCE) using the gold film with hole arrays as an etching catalyst^21^,^22^. The pattern of Au film was duplicated from the anodic aluminum oxide (AAO) template^22^. Figure 3a shows a typical scanning electron microscopic (SEM) image of the Au film on the Si substrate (p-type, resistivity, *ρ* = 1∼10 Ω cm) before MaCE. The Au film-loaded p-type Si substrate was immersed in a mixture solution of hydrofluoric acid (HF) and hydrogen peroxide (H_2_O_2_) (volume ratio; HF:H_2_O_2_ = 1:1) for around 3 min at room temperature (RT) to produce Si NWs. The prepared sample was washed out by deionized (DI) water and was subsequently moved into aqua regia (volume ratio; HCl:HNO_3_ = 3:1) to remove the Au film selectively. Si NWs were cleaned in DI water again. Figure 3b is a plan-view SEM image confirming the formation of vertically-aligned Si NW arrays.

In previous studies, amine groups and glurataldehyde were employed for biomolecule immobilization on silicon surfaces^23^,^24^,^25^. The aligned vertical Si-NW arrays were soaked into piranha solution (volume ratio; H_2_SO_4_:H_2_O_2_ = 3:1) for the passivation of Si NW surface with amine group and glutaraldehyde, thereby forming the hydroxide (-OH)-terminated Si NWs that are easily reactive with amine (-NH_2_) groups, as shown in Fig. 2. The sample was then coated with the 3-aminopropyl triethoxysilane (3-APTES) solution to form the amine-terminated surface. Subsequently, 3-APTES solution was replaced by glutaraldehyde solution to form glutaraldehyde linkers on the surface of Si NWs, which makes it possible for DNAs to be covalently bonded with the surface-modified Si NWs^26^,^27^. For the immobilization of p-ss oligonucleotide on the surface-modified Si NWs, the p-ss oligonucleotide is functionalized with –OH^28^.

### Fabrication of graphene/Si NWs biosensors

For formation of uniform top electrode on Si NWs, poly methyl methacrylate (PMMA)-coated chemical-vapor-deposition (CVD) graphene was transferred onto tips of surface-modified Si NWs, dried by nitrogen gun, and baked on the hotplate at 75 °C for 4 h. The graphene sheet should not cover the entire Si NW tips to secure some part of them, on which the DNA solution can be dropped for DNAs to be spread on the entire surface of Si NWs. The Raman spectrum of graphene on the SiO_2_ substrate shows the D/G ratio of ~0.1 and the G/2D ratio of ~0.47, indicating high-quality monolayer graphene (Supplementary Fig. S1)^29^. Then, the PMMA on graphene was selectively removed in acetone for 1 h. In Fig. 3c, the regions of Si NWs with/without graphene are clearly contrasted. A cross-sectional SEM image in Fig. 3d confirms well-transferred graphene onto the tips of Si NW while uniformly-contacted. Finally, 50-nm-thick Au film was deposited on graphene to form a robust metallic top electrode. The molar concentration of each oligonucleotide solution was controlled from 0.1 to 1000 nM (0.001–10 pmole for 10 μL). 10-μL solutions of the prepared oligonucleotides were dropped onto the surface of the device without the top graphene layer.

### Electrical response

**Figure 4:**
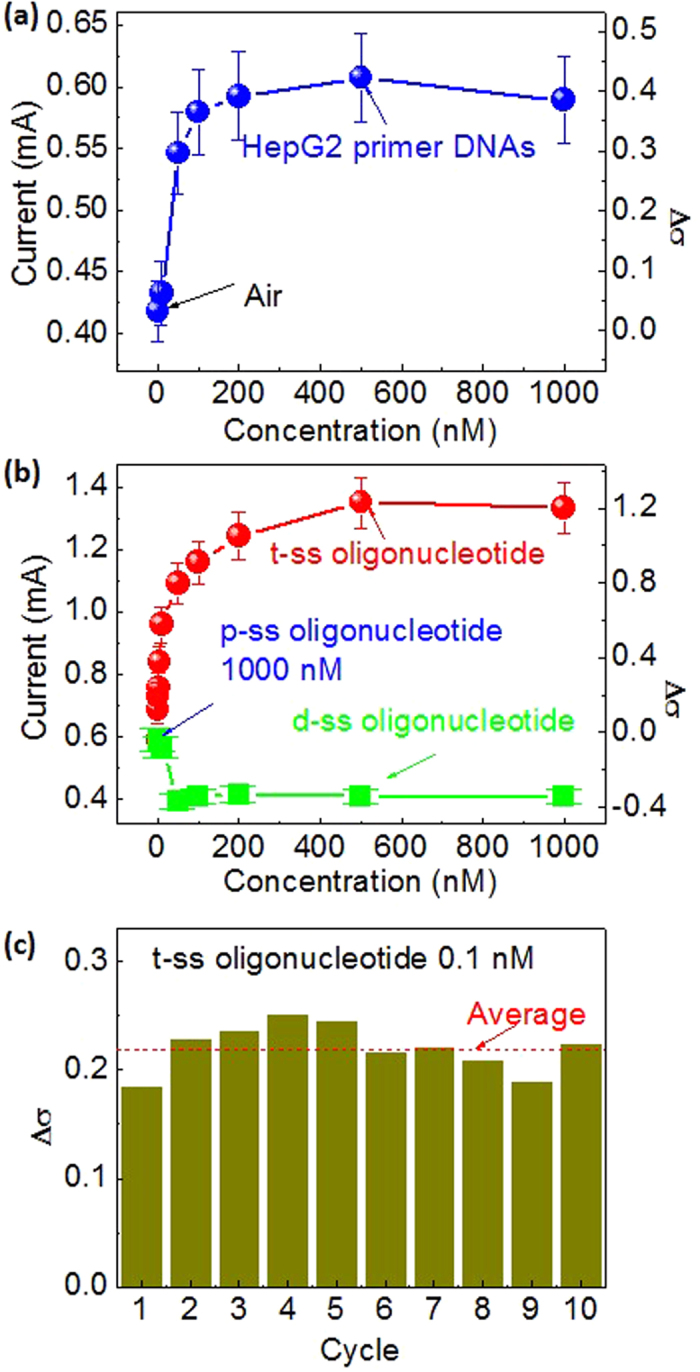
Responsivity of graphene/Si-NWs biosensors. (**a**) Current of a graphene/Si-NWs biosensor as a function of mole fraction of p-ss oligonucleotide. (**b**) Current of a graphene/Si-NWs biosensor as a function of mole fraction of t- or d-ss oligonucleotide loaded on the surface of NWs decorated with 1000 nM p-ss oligonucleotide in advance. The error bars in (**a**,**b**) indicate 6%. (**c**) Endurance histogram for ten-times consecutive sensing of 0.1 nM t-ss oligonucleotide loaded on the surface of NWs decorated with 1000 nM p-ss oligonucleotide in advance. The hybridized p- and t-ss oligonucleotides were well dehybridized and t-ss oligonucleotide was removed without any damage on the device by treatment with sodium chloride.

The graphene/Si-NWs diodes show typical asymmetric *I-V* curves (Supplementary Fig. S2). The conductivity under forward bias is almost twice as large as that under reverse bias, originating from the formation of vertical quasi-Schottky junction between graphene and surface-modified Si NWs (Supplementary Fig. S3). The *I-V* curves were characterized for various molar fractions of each oligonucleotide under a forward bias (Supplementary Fig. S4 for the full forward-biased *I-V* curves). Sensor performances were analyzed at 1 V, which belongs to the active bias region. As shown in Fig. 4a, the current increases gradually as the mole fraction (n_p_) of p-ss oligonucleotide increases from 10 up to 500 nM, and above 500 nM, it saturates. The current saturation might come from almost full coverage of the p-ss oligonucleotide on the surface of Si NWs. We studied the effect of the t-ss oligonucleotide mole fraction (n_t_) on the sensing properties for the device treated by p-ss oligonucleotide at a n_p_ of 1000 nM. For the positive control experiments, the solutions containing t-ss oligonucleotide with various mole fractions were dropped on the device. As shown in Fig. 4b, the device also shows a gradual increase in current as n_t_ varies from 0.1 to 500 nM, and above 500 mM, the current almost saturates, which can be similarly understood as in the variation of n_p_. The current is enhanced by 0.1 mA (~19% increase) even at an extremely-low mole (0.1 pM) of t-ss oligonucleotide, indicating very-sensitive biosensing. The current increases to ~0.76 mA at the saturation point, corresponding to ~120% enhancement. For negative control experiments, solutions containing d-ss oligonucleotide with various mole fractions (n_d_) were dropped on the device. This resulted in the decrease of the current. For example, the current decreases by ~4 and ~30% at n_d_ = 10 and 40 nM, respectively. However, by further increase of n_d_, no significant n_d_ dependence is observed, as shown in Fig. 4b.

The increase of the current can be explained by surface-doping effect of DNAs. When the p-ss oligonucleotide is attached on the surface of Si NWs, the DNA oligonucleotide, acidic bio-species, attract electrons from Si NWs^11^, thereby injecting holes into the valence band of Si NWs. Even when t-ss oligonucleotide is hybridized to p-ss oligonucleotide on the surface of Si NWs, the holes will be also donated into the Si NWs because the hybridization of these oligonucleotides is due to hydrogen bonds formed by base paring without any exchange of charge carriers. As a result, the density of holes in the valence band increases, thereby shifting the Fermi level to the valence band, resulting in the increase of the current through the biosensor. On the other hand, the unhybridized d-ss oligonucleotide is chemically more unstable than the hybridized DNAs, and they can be therefore folded and precipitated onto the unspecific surface of Si NWs. The agglomerated and precipitated d-ss oligonucleotide on the surface of Si NWs can donate a number of electrons to Si NWs, resulting in the decrease of current^30^, as shown in Fig. 4b. The precipitation of d-ss oligonucleotide lump is also confirmed by a PL microscopic image that will be shown below.

The extremely-high sensitivity of the graphene/Si NW biosensors can be understood by following two factors. As one factor, graphene and vertically aligned Si NW arrays act as the efficient carrier collector and the active material, respectively. Graphene can be well contacted with Si NW tips without any other coverage of side surfaces of Si NWs, which makes the sensors more efficient than shown in the previous reports^17^. As another factor, the extremely-high density (~10^9^/cm^2^) and thin diameter (~50 nm) of the Si NWs are very beneficial to the generation of the large charge carriers from the surface of Si NWs.

### Endurance

The endurance properties of the graphene/Si NWs biosensors were studied to check their detection repeatability for the t-ss oligonucleotide. Double stranded oligonucleotide is negatively charged because it possesses the negatively charged phosphate groups. When sodium chloride is added to the solution of double stranded oligonucleotide, the sodium and chloride ions are separated in the solution. Therefore, the positive sodium ions are attached to the negative charged phosphate group of the double stranded oligonucleotide, thereby neutralizing the negative charges. Eventually, the double stranded oligonucleotide is dehybridized, resulting in the generation of the two single stranded oligonucleotides. The hybridized oligonucleotides were detached from the surface of Si NWs by washing out the device in a solution of sodium chloride at 10 mM and subsequently drying it in the air, resulting in denaturation of the double stranded oligonucleotides. 1000 nM p-ss oligonucleotide was then dropped on the device, and the current was measured to check whether the combined double stranded p- and t-ss oligonucleotides were well dehybridized and t-ss oligonucleotide was removed with almost no damage on the device. Subsequently, 0.1 nM t-ss oligonucleotide was dropped to be hybridized to p-ss oligonucleotide on the surface of Si NWs, which resulted in the increase of the current to the level recorded in the previous measurement. These consecutive processes were repeated for ten times. As shown in Fig. 4c, the conductivity change is very uniform with standard deviations ranging ~1 to ~10% through the cyclic experiments for ten times. These results suggest that the endurance of graphene/surface-modified-Si-NWs bio-sensor shows almost negligible degradation even when DNAs with extremely-low molar fraction down to 0.1 nM are detected.

### PL response

**Figure 5:**
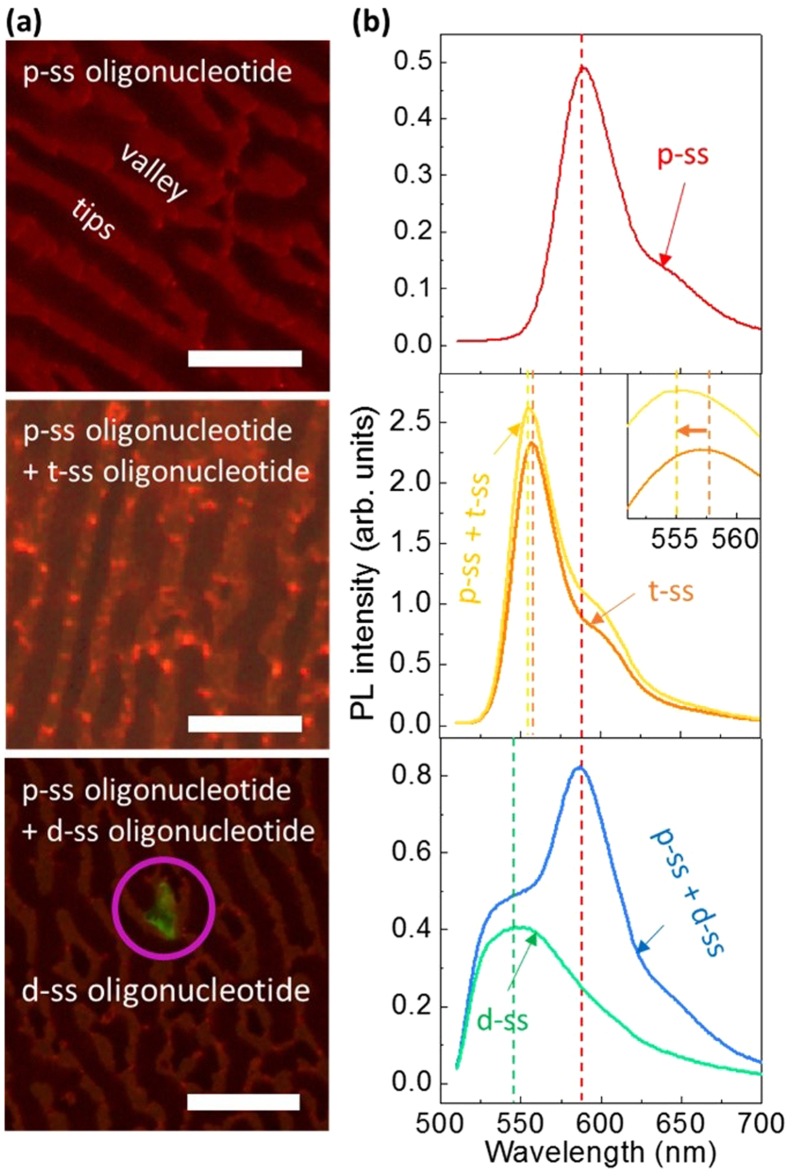
PL microscopic mapping images and spectra of DNA-decorated Si NWs. (**a**) Planar PL microscopic images and (**b**) spectra of Si NWs decorated with p-ss oligonucleotide, p- and t-ss oligonucleotides, and p- and d-ss oligonucleotides. The PL spectra of separate t- and d-ss oligonucleotides are also plotted for the comparisons with those of the hybridized oligonucleotides. The marked area in the PL microscopic image indicates green PL scattered from several spots of Si NWs after d-ss oligonucleotide is added and the inset in the PL spectra clarifies the peak shift. The scale bars indicate 15 μm.

PL microscopy and spectroscopy were performed to check whether the t-ss oligonucleotide is well hybridized to the p-ss oligonucleotide-decorated Si NWs. The p-, t-, and d-ss oligonucleotides have their-own distinguished fluorescence labels of red, orange, and green colors, respectively. For labelling, different fluorophores are attached to the oligonucleotides. Figure 5a shows PL microscopic images of Si NWs decorated with p-ss oligonucleotide only and the hybrids between p-ss oligonucleotide and t- or d-ss oligonucleotide. The p-ss oligonucleotide emits red PL. When t-ss oligonucleotide is added, orange-color PL is clearly observed over whole area. On the other hand, when d-ss oligonucleotide is added, the red-color PL is not changed even though some scattered green PL is observed from several spots of Si NWs, possibly originating from agglomerated and precipitated d-ss oligonucleotide-lumps. This indicates that d-ss oligonucleotide cannot be hybridized to p-ss oligonucleotide.

To clarify the origin of each color from the PL images, the PL spectra were measured for the Si NWs decorated with the three different oligonucleotides species by using a visible laser (λ = 488 nm) as the excitation source. Figure 5b shows PL spectra of solutions containing solely p- oligonucleotide and its mixture with t- or d-ss oligonucleotide. The PL of p-ss oligonucleotide solution is peaked at ~591 nm with a shoulder at ~650 nm, corresponding to a range of red color. The PL of t-ss oligonucleotide solution is peaked at ~557.5 nm with a shoulder at ~600 nm, corresponding to a range of orange color. When the solution of p-ss oligonucleotide is mixed with that of t-ss oligonucleotide, the PL of t-ss oligonucleotide is blue-shifted by ~2.5 nm, as more clearly shown in the inset, together with an enhancement of the shoulder at ~600 nm. To further confirm the slight blue shift, the PL of mixture solution of p- and t-ss oligonucleotides was measured for various mixing ratios (Supplementary Fig. S5). The peak position of PL spectra is gradually blue-shifted as the relative mole fraction of p-ss oligonucleotide increases. These sequential peak shifts seem to be strongly related with the hydridization between p- and t-ss oligonucleotides, possibly resulting in the energy transfer between both kinds of DNAs^30^,^31^,^32^,^33^. However, the PL microscopic image is still seen as orange color because the PL at ~557.5 nm is dominant. On the other hand, the PL of d-ss oligonucleotide solution is peaked at ~547 nm, corresponding to green color. When the solution of p-ss oligonucleotide is mixed with that of d-ss oligonucleotide, two separated PL peaks corresponding to each oligonucleotide are observed without any significant peak shift. These results are consistent with the data of absorption spectra (Supplementary Fig. S6). The absorption peak of the p- and t-ss oligonucleotides mixture is same with that of t-ss oligonucleotide. In contrast, the absorption spectrum of p- and d-ss oligonucleotides mixture shows two separated peaks clearly corresponding to each of them. These results further confirm that dummy oligonucleotide cannot be hybridized to p-ss oligonucleotide.

## Discussion

We demonstrated selectivity, accuracy, and stability of graphene/surface-modified vertical-Si-NWs biosensors for specific oligonucleotides sensing, originating from the unique properties of vertically-aligned Si NWs, including large surface area. The graphene in the biosensors acted as a role of carrier collecting layer on the Si-NW arrays, resulting in the dramatic enhancement of the sensitivity. The p-ss oligonucleotide as DNA receptors and the t-ss oligonucleotide as DNA donors, having complementary nucleotide sequences to the p-ss oligonucleotide, and d-ss oligonucleotide as negative control DNAs, having mismatched nucleotide sequences with those of p-ss oligonucleotide, were employed to detect the DNA-DNA hybridization between specific oligonucleotides with the graphene/Si-NWs diode-type biosensor by analyzing the electrical and PL responses. The biosensors showed the responsivities of 19 to 120% for 0.1-pM to 1000-nM t-ss oligonucleotide, which is remarkably high compared to those previously obtained from single Si-NW device. The excellent reusability of the biosensors was confirmed by ten-times endurance tests. The PL microscopic image and spectra of the DNAs with PL markers also confirmed the mechanism of the selective DNA sensing by the graphene/Si-NWs biosensors.

Our approach utilizes graphene and vertically-aligned Si NW arrays for the building block of the prototypic biosensors, which enables the electrically-sensing DNA devices with outstanding sensitivities to be extremely reduced. The achievement of high-performance graphene/Si-NWs biosensors in view of the detectable molar fraction and the current sensitivity, as compared to the previously-reported graphene-based DNA-sensing performances^18^,^19^, are therefore expected to open exciting opportunities for their diagnostic applications as a portable type or by their integration into the information-processing systems.

## Methods

### Fabrication of Au mesh

The AAO disks were prepared in an electrochemical shell containing an oxalic acid solution at 40 V for 24 hr, and the barrier layer of AAO was then selectively removed in a solution of 5% H_3_PO_4_ for 30 min at 30 °C. The barrier-layer-opened AAO was utilized as the Au-mesh patterning templates. A 25-nm Au film was deposited on the opened barrier-layer side of AAO. Subsequently, the AAO disk was removed in a KOH solution, thereby leaving only the Au mesh with aligned hole arrays. The KOH solution was then replaced to DI water. The Au mesh as catalyst of MaCE was transferred on the p-type Si substrate.

### Preparation of graphene

Large-area graphene layers were grown on 70-μm-thick Cu foils in a graphite-heater-based CVD quartz tube furnace at a growth temperature of 1000 °C with 10-sccm H_2_ and 20-sccm CH_4_ flowing at a pressure of 3 Torr during growth^34^,^35^. The graphene/Cu stack was spin-coated with PMMA, and the Cu was then etched in a nickel etchant for 30 min. The graphene/PMMA stack is then placed in DI water before transferring to the surface-modified Si NW arrays.

### Preparation and handling of oligonucleotide

The synthesized oligonucleotides with specific sequences and fluorescent labels are provided from BIONEER Corporation. The purchased oligonucleotide was dispersed in DI-water of controlled volumes for making p-ss, t-ss, and d-ss oligonucleotide solutions at a molar fraction of 1000 nM, which was further diluted to make the solutions in the range of molar fraction from 0.1 to 500 nM. 10 μL of the prepared solution was taken by using a micropipette, and dropped onto the opened surface of the device without the top graphene layer, so that the solution could be permeated into the gap of surface modified Si NW arrays (Supplementary Fig. S7).

### Measurements

*I-V* measurements to monitor the electrical signals of graphene/Si NWs biosensors were conducted using a Keysight B1500A semiconductor analyzer controlled by an installed program. The probe station was employed for the precise electrical contact on the top- and bottom-electrodes. The PL spectra were measured at RT by using a 488 nm line of Ar ion laser as an excitation source. The PL microscopy was carried out at RT using an epifluorescence microscope (Nikon Instruments). The light beam was focused on the sample surface through a microscope objective with magnifications between 20x and 40x.

## Additional Information

**How to cite this article**: Kim, J. *et al.* Precise and selective sensing of DNA-DNA hybridization by graphene/Si-nanowires diode-type biosensors. *Sci. Rep.* **6**, 31984; doi: 10.1038/srep31984 (2016).

## Supplementary Material

Supplementary Information

## Figures and Tables

**Figure 1:**
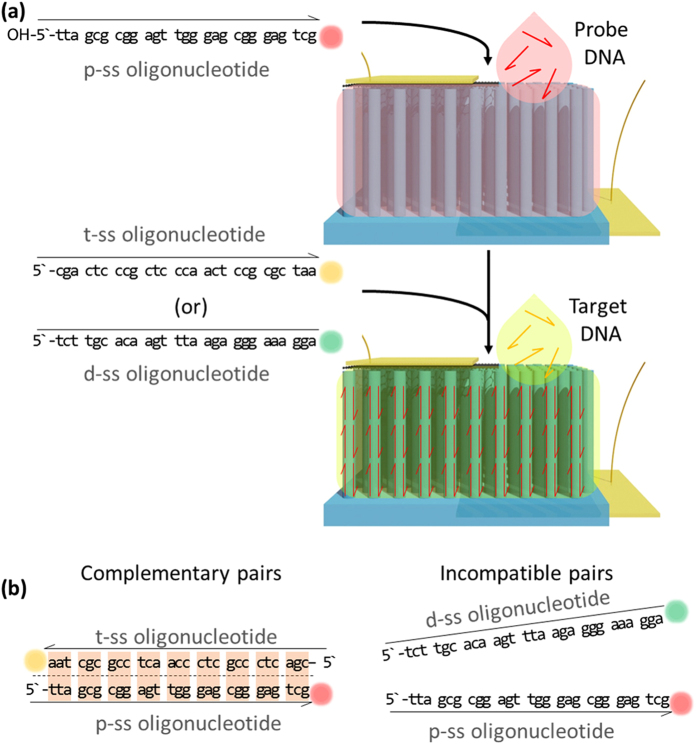
Schematic illustrations describing structure and detecting mechanism of graphene/Si-NW-arrays biosensors. (**a**) Schematic diagram showing the mechanism of graphene/Si-NW-array biosensors for selectively detecting p- and t-, and d-ss oligonucleotides. The Si NWs are decorated with p-ss oligonucleotide in advance for the sensing tests of t- or d- oligonucleotide. (**b**) Schematic diagram of complementary ss oligonucleotide pairs of p- and t-ss oligonucleotides, and incompatible ss oligonucleotide pairs of p- and d-ss oligonucleotides.
