# Supplementary material for: Precise and selective sensing of DNA-DNA hybridization by graphene/Si-nanowires diode-type biosensors
Source: Sci Rep. 2016 Aug 18;6:31984. doi: 10.1038/srep31984 (PMC4989226; doi:10.1038/srep31984)
Supplement: Supplementary Information [file srep31984-s1.doc]

**Supplementary Information**

**for**

**Precise and selective sensing of DNA-DNA hybridization by graphene/Si-nanowires diode-type biosensors**

**Jungkil Kim^1^, Shin-Young Park^3^, Sung Kim^1^, Dae Hun Lee^1^, Ju Hwan Kim^1^, Jong Min Kim^1^, Hee Kang^2^, Joong-Soo Han^3^, Jun Woo Park^1^, Hosun Lee^1^, and Suk-Ho Choi^1*^**

^1^Department of Applied Physics, College of Applied Science, Kyung Hee University, Yongin 446-701, Korea

^2^Department of East-West Medical Science, Graduate School of East-West Medical Science, Kyung Hee University, Yongin 446-701, Gyeonggi-do, Korea

^3^Department of Biochemistry and Molecular Biology, College of Medicine, Hanyang University, Seoul 133-791, Korea

______________________________________________________________

*****To whom correspondence should be addressed. E-mail: sukho@khu.ac.kr

**Figures for Supplementary Information**

**
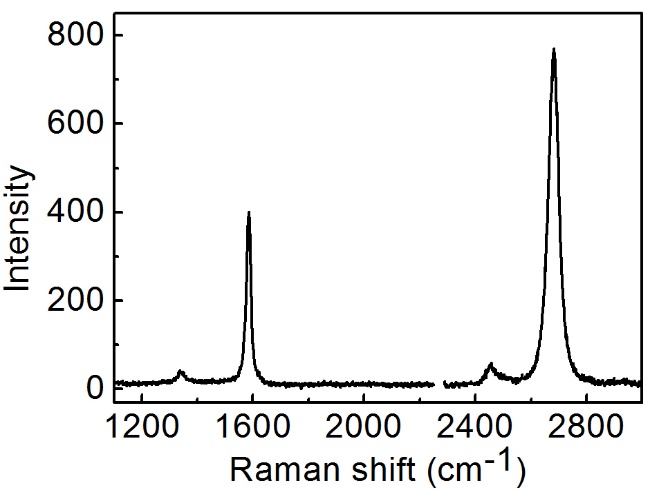
**

**Figure S1.** Typical Raman spectrum of graphene monolayer.

**
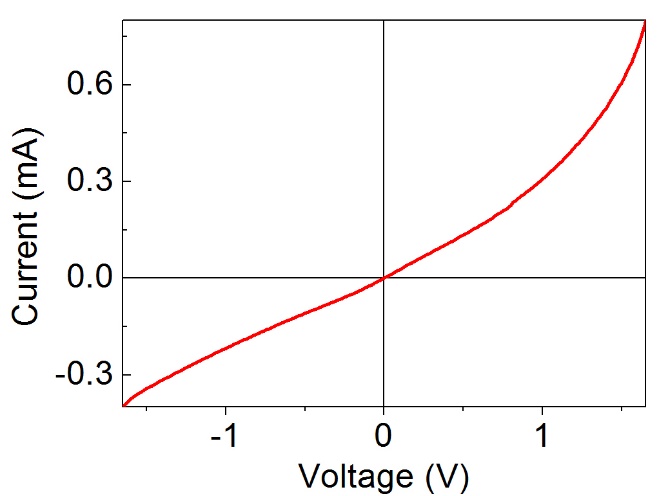
**

**Figure S2.** *I-V* curve of graphene/Si-NWs biosensor. The asymmetric quasi Schottky-diode behavior is observed.


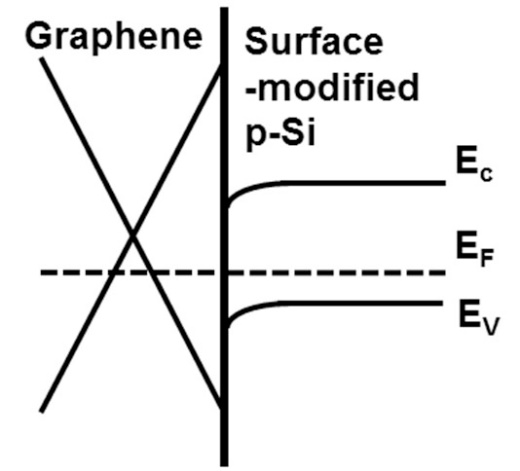


**Figure S3.** Band diagram of graphene/surface-modified Si NWs with a relatively-low barrier height, which enables the current to flow at forward and reverse biases.

**
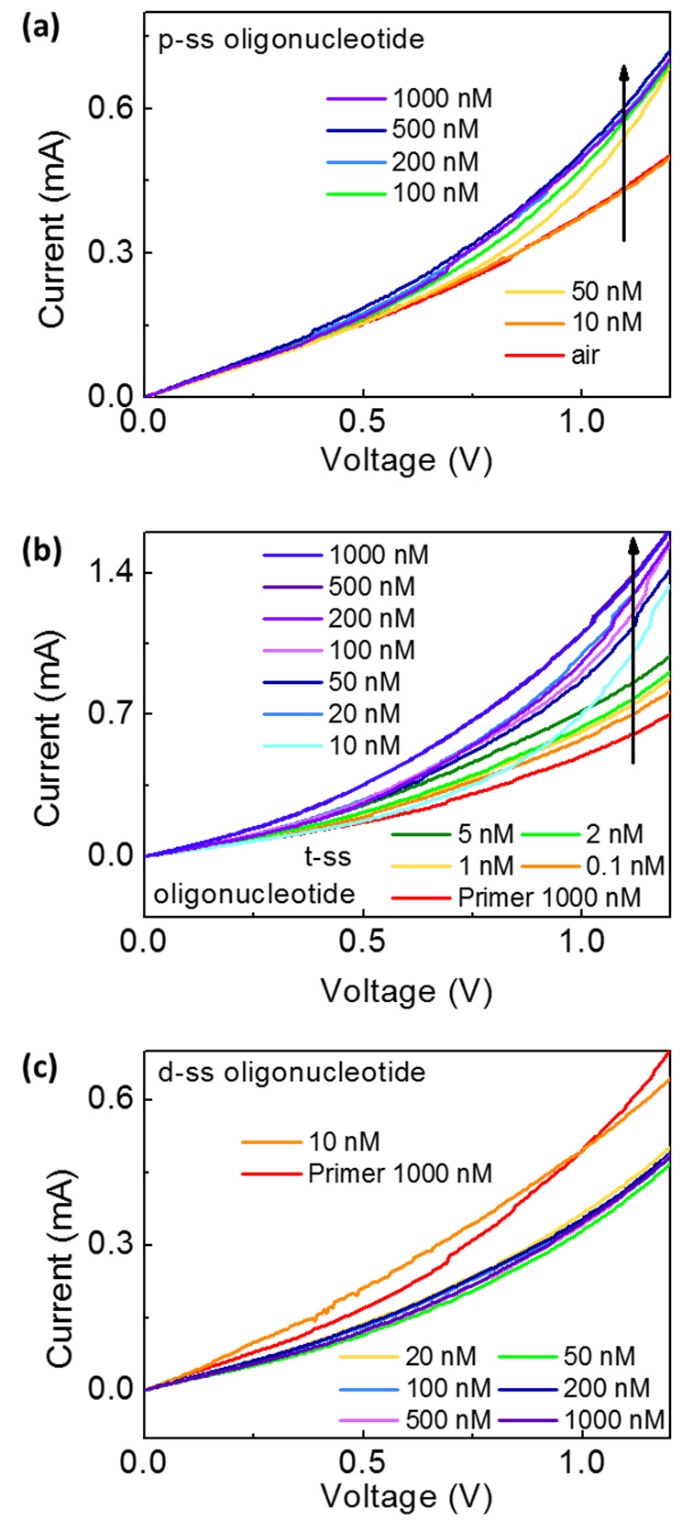
**

**Figure S4.** (a) Full-range *I-V* curves of a graphene/Si-NWs biosensor for various mole fractions of p-ss oligonucleotide. Full-range *I-V* curves of a graphene/Si-NWs biosensor for various mole fractions of (b) t- or (c) d-ss oligonucleotide loaded on the surface of NWs decorated with 1000 nM p-ss oligonucleotide in advance.

**
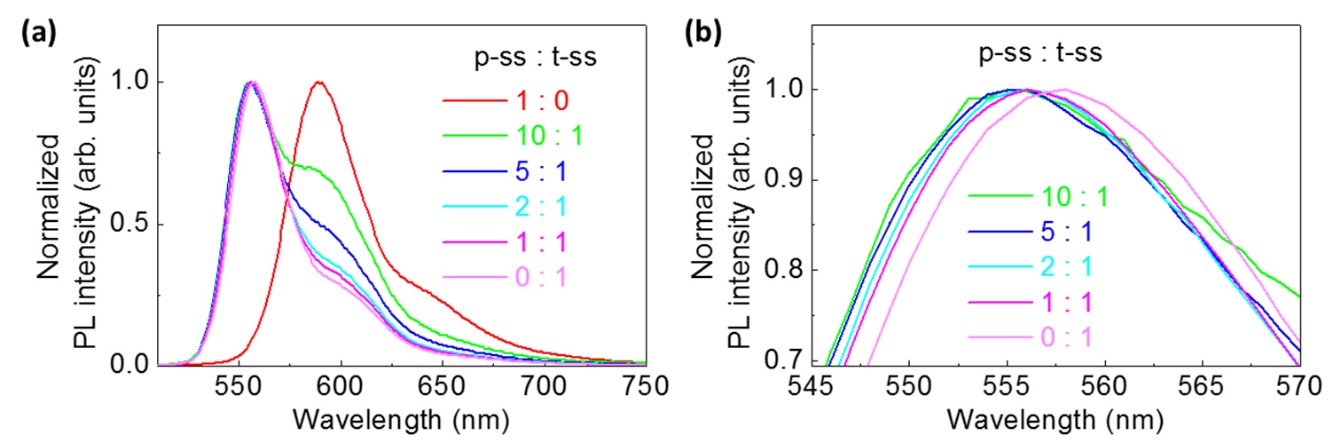
**

**Figure S5.** (a) PL spectra of solutions mixed with p- and t-ss oligonucleotides for various relative ratios of mole fraction. (b) Enlarged PL spectra showing PL peak shifts clearly.

**
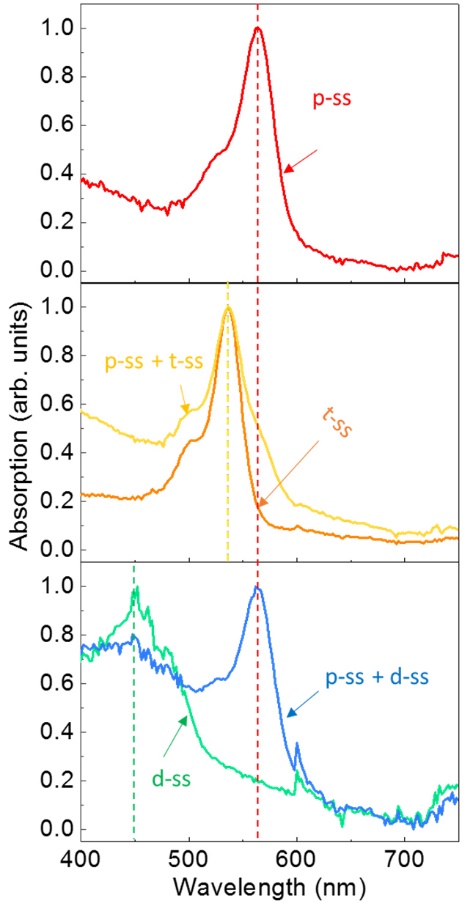
**

**Figure S6.** Absorption spectra of Si NWs decorated with p-ss oligonucleotide, p- and t-ss oligonucleotides, and p- and d-ss oligonucleotides.


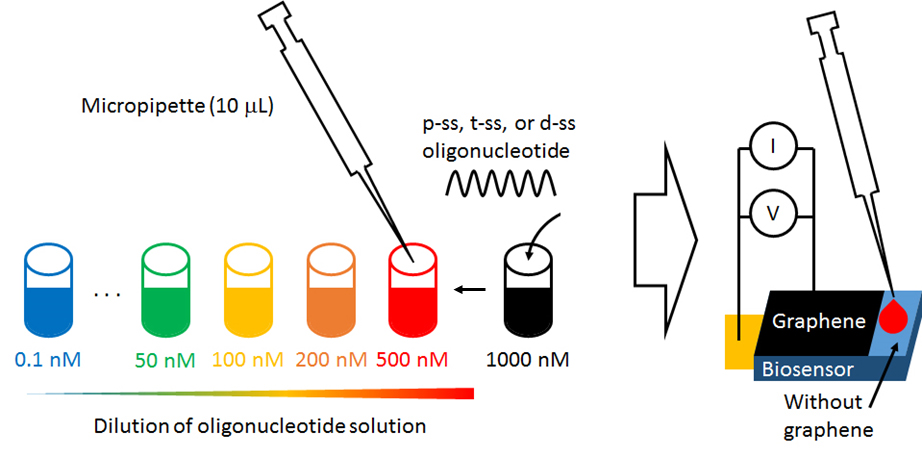


**Figure S7.** Schematic diagrams for showing the sample preparation and experimental setup.
